# Nephrotic syndrome due to preeclampsia before 20 weeks of gestation: a case report

**DOI:** 10.1186/s12882-020-01876-9

**Published:** 2020-06-29

**Authors:** Tomo Suzuki, Daisuke Ichikawa, Mayumi Nakata, Shiika Watanabe, Wei Han, Kaori Kohatsu, Sayuri Shirai, Naohiko Imai, Junki Koike, Yugo Shibagaki

**Affiliations:** 1 Division of Nephrology and Hypertension, Department of Internal Medicine, St. Marianna University School of Medicine, 216-8511, 2-16-1, Sugao, Miyamae-ku, Kawasaki, Kanagawa Japan; 2 Department of Nephrology, Kameda Medical Center, Chiba, Japan; 3 Department of Nephrology and Hypertension, St. Marianna University School of Medicine Yokohama City Seibu Hospital, Yokohama, Kanagawa Japan; 4 Department of Diagnostic Pathology, St. Marianna University School of Medicine, Kawasaki, Kanagawa Japan

**Keywords:** Nephrotic syndrome, Preeclampsia, Soluble fms-like tyrosine kinase-1, Placental growth factor

## Abstract

**Background:**

Preeclampsia (PE) refers to the development of hypertension and new-onset proteinuria or progressive organ damage (especially kidney) in a previously normotensive pregnant women after 20 weeks of gestation. Thus, new-onset nephrotic syndrome due to PE before 20 weeks of gestation seems to be rare, making its diagnosis difficult in this time period.

**Case presentation:**

A 28-year-old woman presented with a new-onset nephrotic syndrome at 16 weeks of gestation. A high dose of oral glucocorticoids (prednisolone, 40 mg) was initiated for presumed glomerulonephritis since she presented with severe nephrotic syndrome before 20 weeks of gestation, however, the treatment was not effective. At 21 weeks of gestation, we confirmed that the soluble fms-like tyrosine kinase-1 (sFlt-1)/placental growth factor (PlGF) ratio was very high (sFlt-1, 13,400 pg/mL; PlGF, 21.9 pg/mL; serum sFlt-1/PlGF ratio 611.9). Therefore, we diagnosed nephrotic syndrome due to PE, and oral glucocorticoids were discontinued. After she underwent a cesarean section at 24 weeks & 3 days, we performed a kidney biopsy. Focal segmental sclerotic lesions with epithelial cell hyperplasia and foam cells in the tubular poles were seen on light microscopy. On immunofluorescence tests, C4d staining showed linear peripheral patterns in the glomeruli. Electron microscopy revealed diffuse subendothelial edema with focal foot process effacement. The histological diagnosis was severe glomerular endotheliosis with focal segmental glomerulosclerosis. Furthermore, the histology of placenta was consistent with PE. Eight months after delivery, her proteinuria disappeared completely.

**Conclusions:**

We not only confirmed an abnormal serum sFlt-1/PlGF ratio but also presented the histology compatible with pure PE in the kidney and placenta in a case of nephrotic syndrome before 20 weeks of gestation. The serum sFlt-1/PlGF ratio may be useful in determining the treatment strategy for atypical cases of pregnant women with nephrotic syndrome, particularly before 20 weeks of gestation.

## Background

Hypertensive disorders of pregnancy (HDP) is a term used to describe increased blood pressure during pregnancy. Preeclampsia (PE) refers to the development of hypertension and new-onset proteinuria or progressive organ damage (especially kidney) in a previously normotensive pregnant women after 20 weeks of gestation. There are various clinical manifestations of the kidney in PE, some of which are acute kidney injury, or proteinuria with or without nephrotic syndrome in PE [1]. In addition, PE has been reported to be associated with chronic kidney disease, including end stage kidney disease [2, 3]. Consequently, the diagnosis of PE is important. However, since the PE is thought to occur after 20 weeks of gestation and the reported cases of PE with nephrotic syndrome before 20 weeks of gestation are rare [4–7], it is difficult to diagnose PE as a causal pathology for nephrotic syndrome and determine treatment strategy for these cases.

Here, we present a case of a woman with nephrotic syndrome developed before 20 weeks of gestation without abnormalities of the fetus. She did not respond to steroid, which is used for presumed glomerulonephritis and was found to have not only abnormal serum levels of soluble fms-like tyrosine kinase-1 (sFlt-1) and placental growth factor (PlGF) as markers of PE, but also the histology of the kidney which was completely compatible with PE after delivery.

## Case presentation

A 28-year-old Japanese woman had infertility, with G5 P1, but not antiphospholipid syndrome. Owing to protein S deficiency, she was treated with aspirin 100 mg per day and heparin therapy, which was discontinued because of a subchorionic hematoma at 12 weeks of gestation. At 16 weeks, she presented with proteinuria and edema in the lower extremities and was transferred to another hospital. A high dose of oral glucocorticoids (prednisolone, 40 mg) was initiated for presumed primary glomerulonephritis before 20 weeks of gestation with severe hypoalbuminemia (1.8 g/dL) and severe proteinuria (10 g/gCre) were suspected. In addition, 40 mg of nifedipine per day and 750 mg of methyldopa hydrate per day were administered for hypertension. Her condition failed to improve, and bilateral pleural effusion appeared. Therefore, she was transferred to our hospital at 21 weeks and 3 days of gestation.

Her medical history was unremarkable, except for infertility. Her birthweight was within the normal range (3260 g). Her height was 156 cm, blood pressure was not high at 128/91 mmHg, and she weighed 53.3 kg. She had remarkable edema in her lower extremities. She did not have sclerodactyly. Laboratory test results were as follows. Serum total protein and albumin levels were very low at 4.9 and 2.2 g/dL, respectively. Serum creatinine and uric acid were slightly high at 0.65 and 6.9 mg/dL, respectively, owing to her gestation. Serum total cholesterol, low density lipoprotein cholesterol, and triglycerides were 510, 329, and 439 mg/dL, respectively. C-reactive protein was 0.37 mg/dL, and urine protein content was 5.6 g/day, with no hematuria. Antinuclear antibody was low titer (1:40), and anti-centromere antibody was positive at 29.3 IU/mL. In addition, anti-dsDNA, anti-β2-glycoprotein I antibody, and anti-phospholipid IgG antibody, lupus anticoagulant were negative. Serum IgG, IgA, C3, and C4 levels were 131, 85, 64, and 10 mg/dL, respectively. Serum IgM and IgE levels were 139 and 133 mg/dL, respectively. Further, protein S activity was in the normal range (95%) in our hospital.

**Fig. 1:**
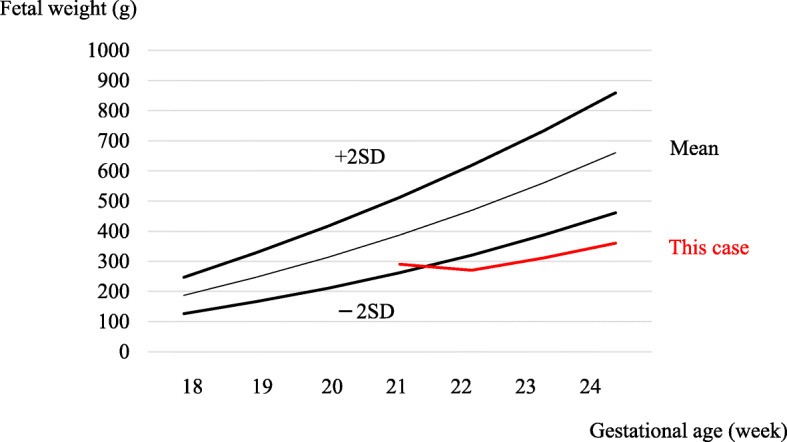
Fetal growth curve from 18 weeks to 24 weeks

**Fig. 2:**
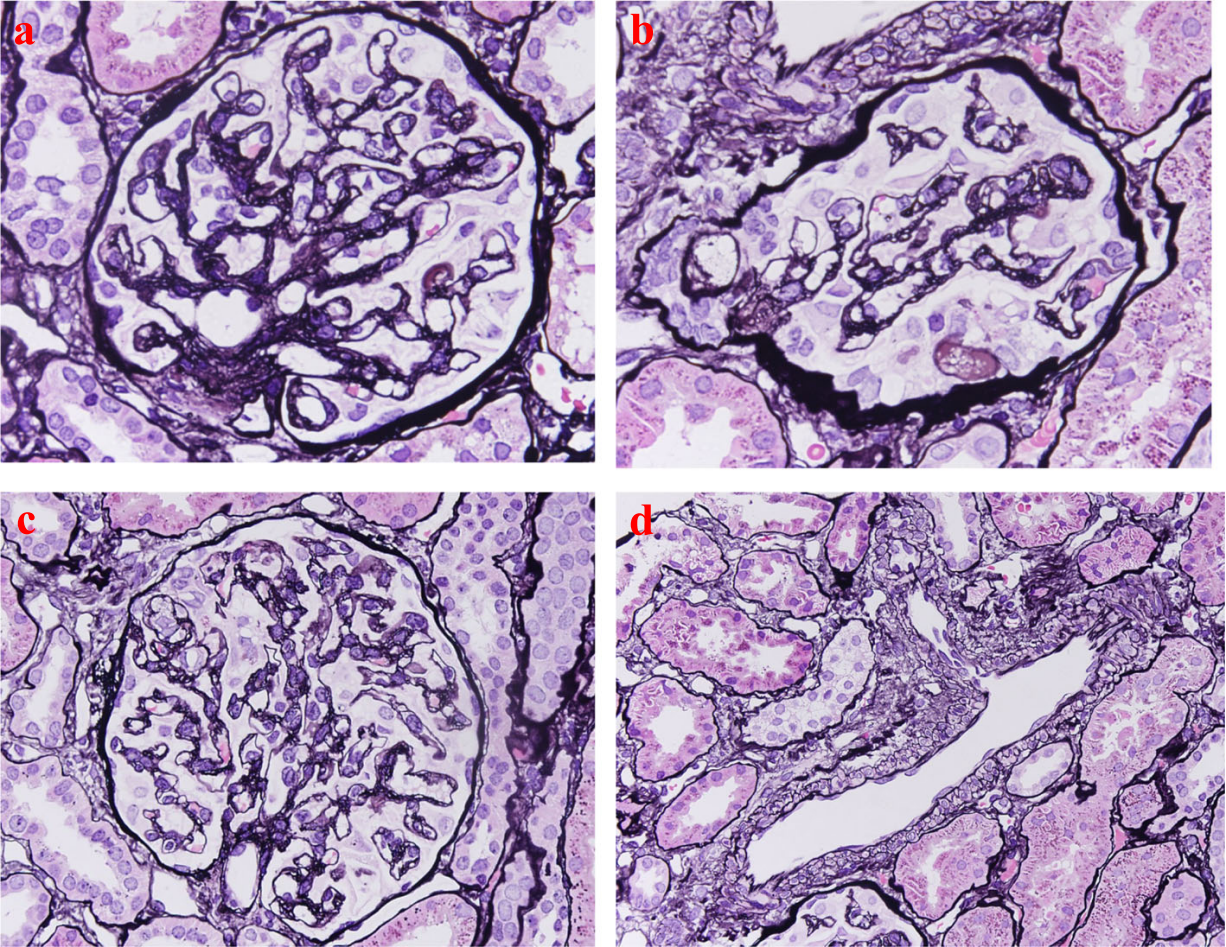
Light microscopy (periodic acid–methenamine silver stain) of the kidney biopsy. **a** In the intracapillary lumina with prominently swollen glomerular endothelial cells, glomerular basement membranes show splitting. **b** This glomerulus is collapsing, with foam cells in the tubular pole lumen and massive hyaline degeneration of podocytes. **c** Severe subendothelial fibrinoid deposits. **d** Swelling is also observed in the endothelial cells of the interlobular arteries

She met the criteria for nephrotic syndrome. However, it was difficult to diagnose primary nephrotic syndrome due to PE before 20 weeks without kidney biopsy. Therefore, we examined serum soluble fms-like tyrosine kinase-1 (sFlt-1) and placental growth factor (PlGF) levels since the ratio of sFlt-1/PlGF (> 36) in pregnant women have been demonstrated as a predictor of PE [8]. The sFlt-1/PlGF ratio turned out to be very high (sFlt-1, 13,400 pg/mL; PlGF, 21.9 pg/mL; serum sFlt-1/PlGF ratio 611.9), so that we diagnosed her nephrotic syndrome because of PE, and we discontinued oral glucocorticoids. We used furosemide and human albumin solution, however, her fetal growth curve was lower than 2 standard deviation after 21 weeks (Fig. 1) and, further, she had a reflux of uterine artery blood flow. Therefore, she had a cesarean section at 24 weeks 3 days. Five weeks after delivery, we performed a kidney biopsy. The core kidney tissue contained 21 glomeruli without global sclerosis. Two of the 21 glomeruli showed focal segmental sclerotic lesions, with epithelial cell hyperplasia and foam cells in the tubular poles (Fig. 2a, b). In addition, spike formations and bubbly appearances of the glomerular basement membrane were absent (Fig. 2c). In addition, hyaline arteriolosclerosis and intimal thickening in the interlobular arteries were not found (Fig. 2d).

**Fig. 3:**
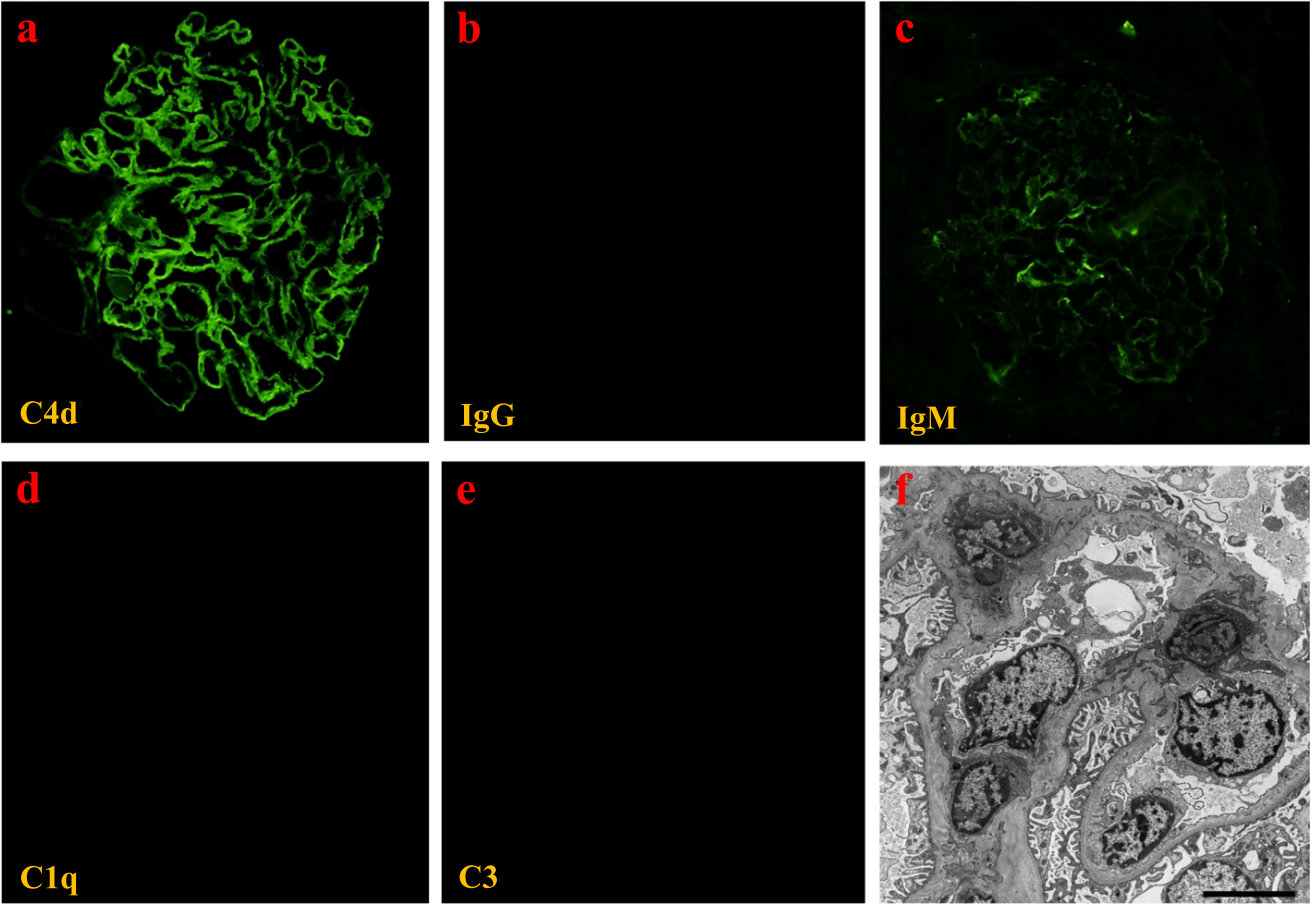
Immunofluorescence results. **a** Image shows glomerular C4d, **b** IgG, **c** IgM, **d** C3 and **e** C1q. **f** Electron microscopy shows prominent glomerular endothelial swelling and no foot process effacement

**Fig. 4:**
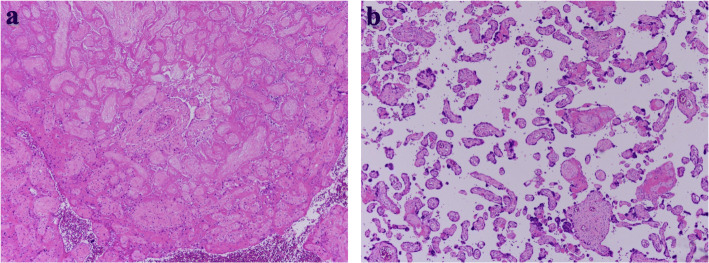
Light microscopy of the placenta (periodic acid stain). **a** Hemorrhagic infarction is seen around the umbilical cord with low-power magnification. **b** Syncytial knots are seen with high-power magnification

**Fig. 5:**
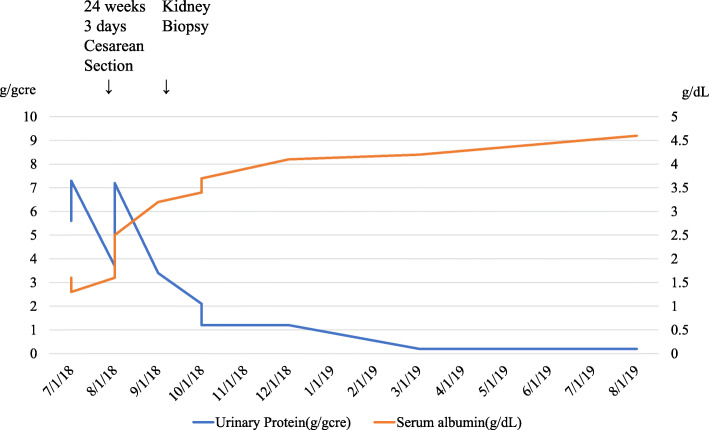
Patient’s clinical course

Immunofluorescence (IF) staining demonstrated diffuse, linear, peripheral patterns of C4d, slight paramesangial patterns of IgM and negative results for IgG, IgA, C3, and C1q. Electron microscopy revealed diffuse subendothelial edema without electron-dense deposits. Further, diffuse foot process effacement was not observed (Fig. 3). The histological diagnosis was severe glomerular endotheliosis with focal segmental glomerulosclerosis (FSGS) lesions. For the histology of the placenta, hemorrhagic infarction around the umbilical cord and syncytial knots were consistent with PE (Fig. 4). As shown in her whole clinical course (Fig. 5), 8 months after delivery, her proteinuria disappeared completely without anti-hypertensive agents, which was also compatible with PE. The neonate was diagnosed with respiratory distress syndrome, rickets, and retinopathy. His weight increased from 303 g to 2806 g 171 days after birth.

## Discussion and conclusions

We present a case of nephrotic syndrome due to PE before 20 weeks of gestation. In addition to her clinical course compatible with PE, we confirmed PE not only by the histology of the kidney and placenta, but also by the serum markers (sFlt-1/PlGF). Further, the risk factors for PE were not clearly identified [9].

PE refers to the development of hypertension and new-onset proteinuria or progressive organ damage (especially kidney) in a previously normotensive pregnant women after 20 weeks of gestation. Therefore, it may not be an appropriate term for nephrotic syndrome before 20 weeks of gestation. Tanaka et al. reported five cases and a literature review [7]; however, reports of nephrotic syndrome associated with pregnancy before 20 weeks are scarce. Hence, we had difficulties in diagnosing this condition. Similar to our case, Tanaka et al. treated nephrotic syndrome using glucocorticoid therapy as an empiric therapy without kidney biopsy. In spite of treatment, urinary protein excretion increased and fell to normal levels within 6 months postpartum [7].

Imasawa et al. showed pure PE with nephrotic syndrome of performing a kidney biopsy at 17 weeks of gestation [5]. In general, it is difficult to decide whether to perform a kidney biopsy on pregnant women with nephrotic syndrome. According to a systematic narrative review, kidney biopsies during pregnancy have a significantly higher risk of complications, which endanger both the mother and fetus, compared to postpartum biopsies [10]. Alternatively, kidney biopsies performed to diagnose glomerulonephritis or PE during pregnancy have led to therapeutic changes in 66% of cases [10]. Therefore, it was difficult to make differential diagnosis for primary nephrotic syndrome without kidney biopsy; PE or glomerulonephritis. Also, we could not perform kidney biopsy because of bilateral pleural effusion in addition to pregnancy. Histological diagnosis is important to determine whether to treat using glucocorticoids without kidney biopsy and interrupt pregnancy before 20 weeks of gestation. Therefore, we selected the treatment strategy based on the sFlt-1/PlGF ratio.

Soluble Flt-1 is known to increase owing to placental ischemia and endometrial vascular endothelial growth factor (VEGF) in PE; further, it neutralizes VEGF [11]. Soluble Flt-1 gradually increases with progression of pregnancy [8]. We confirmed her serum sFlt-1 levels to be 13,400 pg/mL and PlGF to be 21.9 pg/mL at 20 weeks of gestation, however it is difficult to evaluate the absolute serum levels of sFlt-1 and PlGF.

Histologically, the differential diagnosis is FSGS, including primary FSGS. Arteriosclerosis and glomerular hypertrophy in our case were not remarkable. In addition, low glomerular density was unclear because one kidney core included 21 glomeruli. In EM, the rate of foot process effacement was under 50%. Secondary FSGS is commonly characterized by the presence of segmental foot process effacement on EM [12]. Based on the clinical data, she did not have low birthweight. Therefore, we considered that the histological diagnosis was neither primary podocyte disease nor adaptive FSGS. Further, collapsing glomerulopathy, a severe glomerular endothelial injury, often occurs in FSGS lesions [13]. Alejandra et al. reported three cases of collapsing lesions and focal segmental glomerulosclerosis during pregnancy and examined the serum sFlt-1/PlGF ratio in one out of three cases [14]. This ratio was 88.76, and the ratio in our case was much higher than this. Therefore, we considered that the ratio was reflective of the severity in our case. Histological analyses have shown that PE is caused by glomerular endothelial and podocyte injuries [15]. Our case confirmed injuries to not only the endothelial cells, but also the foam cells, as well as segmental sclerosis. For IF, kidney biopsy in our case showed C4d deposits only, not IgG and C1q. C4d deposits may be the result of severe endothelial injury, such as antibody mediated rejection in kidney transplantation. Recently, C4d deposits are frequently seen owing to classical complement pathway activation in the kidney of preeclamptic women [16]. Considering the histology and clinical findings, our patient’s nephrotic syndrome was diagnosed as pure PE.

In conclusion, we present a case of a woman with nephrotic syndrome developed before 20 weeks of gestation due to PE, which was confirmed by typical clinical course, abnormal raio of soluble fms-like tyrosine kinase-1 (sFlt-1)/ placental growth factor (PlGF) and the histology of the kidney and placenta. The serum sFlt-1/PlGF ratio may be very useful and significant in the diagnosis of PE in pregnant women with nephrotic syndrome, particularly before 20 weeks of gestation.

## Data Availability

The datasets used and/or analyzed during the report are available from the corresponding author on reasonable request.

## Abbreviations

EM: Electron microscopy
FSGS: Focal segmental glomerulosclerosis
IF: Immunofluorescence
PE: Preeclampsia
PIH: Pregnancy-induced hypertension
PlGF: Placental growth factor
VEGF: Vascular endothelial growth factor
sFlt-1: soluble fms-like tyrosine kinase-1
